# Prophylactic use of dural tenting sutures in elective craniotomies in adults—is it necessary? A study protocol for a multicentre, investigator- and participant-blinded randomised, parallel-group, non-inferiority trial

**DOI:** 10.1186/s13063-021-05201-z

**Published:** 2021-04-12

**Authors:** Przemysław Kunert, Łukasz Przepiórka, Jan Fortuniak, Karol Wiśniewski, Ernest Jan Bobeff, Patrycja Larysz, Rafał Kruk, Bartłomiej Kulesza, Dariusz Szczepanek, Piotr Ładziński, Jarosław Żyłkowski, Sławomir Kujawski, Kamila Łabędzka, Dariusz Jaskólski, Radosław Rola, Tomasz Trojanowski, Andrzej Marchel

**Affiliations:** 1 Department of Neurosurgery, Medical University of Warsaw, Banacha St. 1a, 02-097 Warsaw, Poland; 2 Department of Neurosurgery and Oncology of Central Nervous System, Barlicki University Hospital, Medical University of Lodz, Lodz, Poland; 3 Department of Neurosurgery, Medical University of Silesia, Regional Hospital, Sosnowiec, Poland; 4 Neurosurgery and Pediatric Neurosurgery Department in Lublin, Medical University of Lublin, Lublin, Poland; 5 Second Department of Radiology, Medical University of Warsaw, Warsaw, Poland; 6 Department of Hygiene, Epidemiology and Ergonomics, Collegium Medicum in Bydgoszcz, Nicolaus Copernicus University in Toruń, Toruń, Poland; 7 Medical University of Warsaw, Warsaw, Poland

**Keywords:** Dural tenting sutures, Craniotomy, Extradural haematoma

## Abstract

**Background:**

In the early days of neurosurgery, extradural haemorrhages (EDHs) contributed to a high mortality rate after craniotomies. Almost a century ago, Walter Dandy reported dural tenting sutures as an effective way to prevent postoperative EDH. Over time, his technique gained in popularity and significance to finally become a neurosurgical standard. Yet, several retrospective reports and one prospective report have questioned the ongoing need for dural tenting sutures. Dandy’s explanation that the haemostasis observed under hypotensive conditions is deceiving and eventually causes EDH may be obsolete. Today, proper intra- and postoperative care, including maintenance of normovolemia and normotension and the use of modern haemostatic agents, may be sufficient for effective haemostasis. Thus, there is a fundamental need to evaluate the necessity of dural tenting sutures in a solid, unbiased, evidence-based manner.

**Methods:**

This study is designed as a randomised, multicentre, double-blinded, controlled interventional trial with 1:1 allocation. About one half of the participants will undergo craniotomy without dural tenting sutures and will be considered an intervention group. The other half will undergo craniotomy with these sutures. Both groups will be followed clinically and radiologically. The primary outcome is reoperation due to extradural haematoma. Secondary outcomes aim to evaluate the impact of dural tenting sutures on mortality, readmission risk, postoperative headaches, size of extradural collection, cerebrospinal fluid leak risk and the presence of any new neurological deficit. The study protocol follows the SPIRIT 2013 statement.

**Discussion:**

It is possible that many neurosurgeons around the globe are tenting the dura in elective craniotomies which brings no benefit and only extends the operation. Unfortunately, there is not enough data to support or reject this technique in modern neurosurgery. This is the first study that may produce strong, evidence-based recommendations on using dural tenting sutures.

**Trial registration, ethics and dissemination:**

The Bioethics Committee of the Medical University of Warsaw approved the study protocol (KB/106/2018). The trial is registered at http://www.clinicaltrials.gov (NCT03658941) on September 6, 2018. The findings of this trial will be submitted to a peer-reviewed neurosurgical journal. Abstracts will be submitted to relevant national and international conferences.

**Trial status:**

Protocol version and date: version 1.5, 14.01.2020

First recruitment: September 7, 2018

Estimated recruitment completion: September 1, 2021

**Table:** 

Data category	Information
Primary registry and trial identifying number	ClinicalTrials.gov NCT03658941
Date of registration in primary registry	6 September, 2018
Secondary identifying numbers	KB/106/2018
Source(s) of monetary or material support	Medical University of Warsaw
Primary sponsor	Medical University of Warsaw
Secondary sponsor(s)	N/A
Contact for public queries	Przemyslaw Kunert MD, PhD tel.: 22-599-25-29
Contact for scientific queries	Łukasz Przepiorka przepiorka@mp.pl
Public title	Dural Tenting Sutures in Neurosurgery - is it Necessary?
Scientific title	Prophylactic Use of Dural Tenting Sutures in Elective Craniotomies - is it Necessary? A Multicentre Randomised Study
Countries of recruitment	Poland
Health condition(s) or problem(s) studied	extradural haematoma
Intervention(s)	No dural tenting techniques
Key inclusion and exclusion criteria	male or female over 18 and under 75 years old qualified for an elective supratentorial craniotomy with a diameter of at least 3 cm Glasgow Coma Scale 15 preoperatively Modified Rankin Scale 0, 1 or 2 preoperatively
Study type	Interventional Allocation: randomised Intervention model: parallel assignment Masking: double blind (subject, investigator) Primary purpose: prevention
Date of first enrolment	7 September, 2018
Target sample size	2 000
Recruitment status	Recruiting
Primary outcome(s)	Reoperation due to extradural haematoma
Key secondary outcomes	Postoperative 30-day mortality Postoperative 30-day readmission to a neurosurgical or neurological department New neurologic deficit or deterioration of a previous one as evaluated on postoperative day 5–7. Cerebrospinal fluid leak requiring treatment. Deterioration of postoperative headaches measuring >5 on the Numerical Rating Scale Extradural collection thickness >3 mm measured radiographically Midline shift >5 mm

World Health Organisation Trial Registration Data Set

*N/A* not applicable

**Date and version identifier:**

14.01.2020, version 1.5

**Table:** 

Date	Version
20.06.2018	Original
13.09.2018	Amendment 01: Control CT scan between 1 and 3 days postoperatively
05.10.2018	Amendment 02: Second control evaluation between 5 and 7 days after the surgery, or earlier if subject is to be discharged earlier
20.11.2018	Amendment 03: Secondary outcome “Extradural collection volume >10 ml measured radiographically” has been removed
23.11.2018	Amendment 04: Recruitment status has been changed: all participating sites are enrolment by invitation
14.01.2020	Amendment 05: Study locations and status have been updated (Szczecin and Bydgoszcz were added).

Revision chronology

## Introduction

In the early days of neurosurgery, extradural haemorrhages (EDHs) contributed to a high mortality rate after craniotomies. Almost a century ago, Walter Dandy reported dural tenting sutures as an effective means of preventing postoperative EDH [1]. The sutures elevate the dura, a layer between the brain and the skull, and are thereby thought to prevent EDH formation by eliminating the potential space for blood collection. Over time, Dandy’s technique gained popularity and became a neurosurgical standard.

Yet, there have been several retrospective and prospective reports that questioned the ongoing need for dural tenting sutures [2–4]. Apparently, Dandy’s explanation that the haemostasis observed under hypotensive conditions is deceiving and eventually causes EDH may be obsolete. These days, proper intra- and postoperative care, including maintenance of normovolemia and normotension and the use of modern haemostatic agents, may be sufficient for effective haemostasis. Evasion of this suturing technique by some surgeons supports this argument even further. Moreover, there has also been one randomised clinical trial that neglected the need for Dandy’s tenting sutures [5]; however, its design included several flaws. For example, most of the patients underwent emergency craniectomies, which impeded quantitative evaluation of extradural collection and the midline shift that caused.

Dural tenting sutures not only prolong the surgical procedure, but also potentially increase the risk of complications such as cerebrospinal fluid (CSF) leakage caused by puncturing the dura. Furthermore, the needle of the suture could also damage cortical matter, or superficial cortical vessels underlying the dura, with subsequent subdural or intracerebral haemorrhage. On the other hand, not tenting the dura may increase the risk of EDH formation, as it has never before been evaluated in a solid study. Nevertheless, it is worth mentioning that dural tenting sutures do not protect against EDH in an absolute manner.

In addition, any fluid or gas collection in the extradural space, including EDH, causes stretching of the dura mater. Tension of this innervated structure may cause postoperative headaches. Conversely, dural tenting sutures, by definition, pull up the dura, and theoretically may also contribute independently to headaches. In summary, the impact of dural tenting sutures on postoperative headaches remains unknown, because it has never before been investigated in the literature. Thus, there is a fundamental need to evaluate the necessity of dural tenting sutures in a solid, unbiased, evidence-based manner [6]. As such, it is an example of an Evidence Reversal study [7].

### Trial objectives and hypothesis

The main objective of this trial is to assess the necessity of prophylactic use of dural tenting sutures in elective, supratentorial craniotomies. The null hypothesis of this study is that the risk of EDH formation when the dura is not tented is higher than when the dura is tented (‘non-inferiority study’).

Not tenting the dura will be considered as an intervention. Consequently, the control group will consist of patients with dural tenting sutures. In this study, one half of the randomly assigned participants will undergo craniotomy without dural tenting sutures and will be considered an intervention group. The other half will undergo craniotomy with these sutures.

### Study design

This study is designed as a randomised, multicentre, investigator- and participant-blinded, controlled interventional trial with 1:1 allocation and is described in detail below. The study protocol follows the SPIRIT 2013 statement [8]. At the time of preparation of this paper, there are six participating centres; however, it is possible to add more if needed.

## Methods

The trial is registered at http://www.clinicaltrials.gov (NCT03658941), and any important changes in the protocol will be implemented there (World Health Organisation Trial Registration Data Set).

### Setting and participants

Study recruitment will take place in Poland, in six participating neurosurgical departments: Department of Neurosurgery, Medical University of Warsaw, Warsaw, Poland; Department of Neurosurgery and Oncology of Central Nervous System, Barlicki University Hospital, Medical University of Lodz, Lodz, Poland; Department of Neurosurgery, Medical University of Silesia, Regional Hospital, Sosnowiec, Poland; Neurosurgery and Pediatric Neurosurgery Department in Lublin, Medical University of Lublin, Lublin, Poland; Department of Neurosurgery, 10th Military Research Hospital and Polyclinic, Bydgoszcz, Poland; Department of Neurosurgery and Pediatric Neurosurgery, Pomeranian Medical University, Szczecin, Poland. Patients meeting the inclusion criteria will be invited to take part in the study by the study investigators performing study in each centre. Each patient qualified for an elective, supratentorial craniotomy should undergo screening. Two thousand subjects are planned to be included in the study, according to the statistical evaluations. Each study-eligible subject will be assigned in random order to an intervention or control group and, subsequently, will undergo a supratentorial craniotomy for unrelated pathology (brain tumours, vascular malformations, etc.). Both groups will be followed radiologically and clinically in the exact same manner, as detailed in the text.

### Inclusion criteria

Adults eligible for the trial must fulfil all of the following criteria:

- Male or female over 18 and under 75 years old;
- Qualified for an elective supratentorial craniotomy with a diameter of at least 3 cm;
- Glasgow Coma Scale 15 preoperatively;
- Modified Rankin Scale 0, 1 or 2 preoperatively.

### Exclusion criteria

Any patient will be excluded in case of:

- Coagulation abnormalities before the surgery;
- Revision craniotomy;
- Skull base surgery.

The latter criterion was added because skull base surgery is significantly different from a supratentorial approach, and currently, there is not enough data to support safe testing of dural tenting in skull base surgery. The common pterional craniotomy was at least partly considered skull base surgery and therefore excluded. Except for those mentioned above, there are no relevant concomitant care or interventions that are prohibited during the trial.

Revision surgeries are not included because previous placement (or lack of placement) of dural tenting sutures might impact the current allocation.

### Statistical analysis, sample size, recruitment and allocation

Sample size calculation was made using calculator available on-line (Sealed Envelope Ltd. 2012. Power calculator for binary outcome non-inferiority trial. [Online] Available from: https://www.sealedenvelope.com/power/binary-noninferior/ [Accessed Mon Sep 23 2019]). Alpha level of 0.05, power (1-beta) 90% were set; 0.7% EDH occurrence in the experimental group, while in control group expected frequency of 1.4% was assumed. Non-inferiority limit, *d*, was set on 0.7%. The study aims to include 908 patients in every study group, 1816 patients in total. Because a loss of about 10% (181.6) of patients is expected due to nonadherence of the surgeons to the allocation and rounding up by 2.4, 1000 patients will be included in each group, 2000 patients in total. However, the risk of extradural haematoma may be evaluated more accurately after a systematic review is prepared regarding the necessity of dural tenting sutures, resulting in possible change of sample size.

Each eligible patient will be invited to take part in the study. Informed consent will be obtained from study investigators, who are also the surgeons performing the operation. All patients who give consent for participation and who fulfil the inclusion criteria will be randomised.

The allocation sequence with the block size of 100 for each participating centre was generated by a professional statistician using a computer-generated consecutive list for either intervention or control. Each consecutive participant is assigned a randomisation number, specific to each centre, which has had already been allocated to the intervention or control group. Randomisation numbers are assigned in a growing fashion (i.e. 1, 2, 3, 4...). Statistical methods for analysing primary and secondary outcomes and all other relevant details can be found in the s Statistical Analysis Plan (SAP) at http://www.clinicaltrials.gov.

### Interventions

Because dural tenting has been regarded as a neurosurgical standard for decades, not tenting the dura will be considered an intervention. The control group will consist of patients with at least 3 dural tenting sutures near the edge of the opening to the bone or pericranium. Central tenting sutures will be regarded separately and data about them will also be collected if these were to be used in the control group. The minimum number was selected arbitrarily, but seems reasonable as a minimal number to consider dural tenting.

Non-adherence to the allocation is possible in either group should the patient’s safety be compromised. One such circumstance is the occurrence of excessive extradural bleeding that cannot be stopped using standard haemostatic agents in a patient allocated to an intervention group. Conversely, dural tenting sutures cannot be placed in a control group subject should the dura be torn apart along the borders of the craniotomy. Any deviation from the allocation must and will be reported by the surgeon in the randomisation chart dedicated to each participant, which will be discussed in the next section of the text. No strategies to improve adherence to the intervention protocols will be implemented due to the nature of the surgical study and safety of the participants.

### Randomisation and allocation

Each participant will be assigned a randomisation chart, which will include the allocation information of the participant and detailed allocation instructions. The surgeon is supposed to open the envelope containing the randomisation chart shortly before the operation.

### Randomisation chart

Upon finishing the surgery, the surgeon will complete all the information requested in the randomisation chart and seal completed chart back in the envelope. The information provided postoperatively by the surgeon in the randomisation chart includes:

- Water tightness of the closure of the dura;
- Usage of any wound drains;
- Occurrence of excessive bleeding from the edges of the craniotomy (from the diploe or extradural space);
- Number of dural tenting sutures placed at the edge of the craniotomy (as originally described by Dandy);
- Number of dural tenting sutures placed at the centre of the dural flap (as originally described by Poppen [9]);
- List of all haemostatic agents used;
- A description of the reason for any change of allocation (if applicable).

### Clinical evaluation

The clinical evaluation schedule consists of 3 short neurological examinations, according to the trial schedule (Table 1). The first examination is to take place 1 day before the surgery, the second is 1 day after surgery and final is between 5 and 7 days after the surgery (or earlier if the subject is to be discharged earlier). At each neurological examination, patients will be evaluated according to the Glasgow Coma Scale (GCS) and modified Rankin Scale (mRS), their headaches will be assessed according to the numerical rating scale (NRS) and the muscle power in each limb will be assessed according to the modified Research Council System.

**Table 1:** Trial schedule

	Study period						
	Enrolment	Allocation	Post-allocation				Close-out
**Time point**	***Day before the surgery***		***Day of the surgery***	POD 1	POD 1–3	POD 5–7^*^	POD 30
**Enrolment:**							
**Eligibility screen**	X						
**Informed consent**	X						
**Allocation**	X						
**Interventions:**							
***Intervention group***			X				
***Control group***			X				
**Assessments:**							
***Basic demographic data acquisition***	X						
***Neurological evaluation***	X			X		X	
***CT***					X		
***Mortality and readmission evaluation***							X
***Radiological evaluation of a CT imaging***							X

*Abbreviations*: *POD* postoperative day, *CT* computed tomography, *NRS* Numerical Rating Scale, *GCS* Glasgow Coma Scale, *mRS* modified Rankin Scale, *MRCs* Modified Research Council system, *DOB* date of birth

*Or earlier if the patient is discharged before POD 5

### Radiologic evaluation

The radiologic evaluation will include the most recent preoperative imaging [either magnetic resonance imaging (MRI) or computed tomography (CT)] and the postoperative CT completed according to the trial schedule (Table 1). During preoperative imaging, the measurement will include the midline shift; postoperative CT measurements will include the maximal thickness of the extradural collection, the size of the bone opening and the midline shift.

### Assessments and data collection

Data collection methods include mostly basic clinical and radiologic evaluations, all of which will be performed by trained and experienced doctors. Additionally, standardised training and testing of clinical outcome assessors will be performed to ensure reliability of the results. However, all radiologic data will be assessed by a central committee consisting of two independent radiologists. To do so, anonymised CT scans from all centres will be transferred to the main centre for subsequent storage and evaluation. All data will be entered manually on paper case report forms (CRFs) and subsequently collected electronically for final evaluation. All participant files will be stored in numerical order and stored in a secure study site in each study centre. All study-related information will be stored securely at the study site. All participant information will be stored in locked file cabinets in areas with limited access and will be identified by a randomisation code. Each file will be stored for 3 years following completion of the trial.

Relative risk will be applied to examine primary outcome. In order to show the possible impact of a lack of data on the results, ‘intention to treat’ (all randomised participants, irrespective of protocol adherence), ‘per protocol’ (only those participants, who were treated according to the protocol) and ‘as treated’ (all participants according to the treatment they received) analyses will be performed.

### Subject participation and follow-up

Once a patient is enrolled or randomised, the study site will make every reasonable effort to follow the participant for the entire study period. Participants may withdraw from the study for any reason at any time. Furthermore, this type of study, evaluating primarily reoperation due to postoperative extradural haematoma, does not require any long-term follow-up because EDH is a short-term postoperative complication. Nevertheless, the overall 30-day mortality among participants will still be evaluated.

### Interim monitoring and data monitoring committee

According to the decision of the Bioethics Committee of the Medical University of Warsaw (TBC-MUW), the study blind will be broken once after the enrolment of the first 100 subjects, and interim monitoring will be conducted with the purpose of establishing the safety of the trial. TBC-MUW is independent of the study organisers. In light of this interim analysis, TBC-MUW will independently advise whether to stop or continue the study.

### Harms and auditing

The study will monitor for CSF leak, extradural haematoma and other adverse events daily through patient examination by an investigator designated for a specific centre. The CRF contains meticulous description of all potential adverse events with clinical information to be provided related to the specific event. Severe adverse events will be reported to TBC-MUW. There is no external auditing planned for this study. However, periodic controls for other than coordinating centres will be performed by principal investigators.

Patients that are enrolled in the study are covered by indemnity for negligent harm through the standard National Health Found Indemnity arrangements.

### Allocation concealment and blinding

Due to the nature of the surgical procedures, the surgeon and the rest of the operating room (OR) medical team will be aware of the current subject’s allocation. However, in each case, the specific OR team aware of the subject’s allocation will be different from the investigators evaluating the given subject. Specifically, in each case, data in the CRF will be completed by an investigator who did not participate in the surgery at any point. Thus, study participants, investigators and outcome assessors will be blinded. The following study procedures will be in place to ensure double-blind administration of the study:

- Access to the randomisation code will be strictly controlled;
- The surgeon will receive information on each subject’s allocation after the surgery has commenced in a sealed envelope containing the allocation information of the participant and detailed allocation instructions.

The study blind will be broken:

1. During interim monitoring, after recruiting the first 100 patients.
2. On completion of the clinical study and after the study database has been locked.
3. When any patient’s safety requires access to the allocation data.

### Primary outcome

The primary outcome is the risk of reoperation due to extradural haematoma (EDH occurrence). This endpoint, as opposed to overall mortality, was chosen because of the main objective of trial, which is to assess the necessity of prophylactic use of dural tenting sutures. The main reason to use such sutures is to prevent reoperation due to EDH; therefore, the primary outcome should directly evaluate that. This outcome will be evaluated during the postoperative hospitalisation of each patient.

### Secondary outcomes

1. Postoperative 30-day mortality

The data to measure postoperative 30-day mortality will be obtained from a national database 30 days after the recruitment of all participants has been completed. Time frame: 30 postoperative days. Descriptive methods: percentage and number of occurrence with confidence intervals. Mean and median value with CI where applied.

2. Postoperative 30-day readmission to a neurosurgical or neurological department

The data required to evaluate readmission rates will be obtained from the hospital databases. Time frame: 30 postoperative days.

3. New neurologic deficit or deterioration of a preoperative deficit, as evaluated on postoperative day 5–7. Time frame: during hospitalisation, as evaluated 5–7 days postoperatively, or earlier if the patient is discharged before the fifth postsurgical day.
4. Cerebrospinal fluid leak requiring treatment. Time frame: during hospitalisation, 5–7 days postoperatively, or earlier if patient is discharged before the fifth postsurgical day.
5. Increase of intensity of postoperative headaches over 5 Numerical Rating Scale from baseline. Time frame: 5–7 days postoperatively, or earlier if the patient is discharged before the fifth postsurgical day.
6. Extradural collection thickness > 3 mm, measured radiographically. Time frame: to be performed shortly after the images are obtained.
7. Midline shift > 5 mm, measured radiographically. Time frame: to be performed shortly after the images are obtained.

### Time schedule

Enrolment of participants was planned to begin on September 1, 2018, in the coordinating centre, and will be finished on September 1, 2021, or earlier, when 2000 participants have been gathered; this is the estimated number of participants needed to achieve study objectives.

### Subgroup analysis

Except for primary and secondary outcomes, certain parameters will be collected prospectively, including data from the randomisation chart and evaluation of craniotomy size. The arbitrary diameter cutoff for a craniotomy to be considered ‘large’ was chosen as ≥ 8 cm. In addition, any deviation from the primary allocation will be carefully and statistically evaluated.

### Final trial dataset, authorship and data sharing statement

The full dataset will be available for all principal investigators with no restriction of access. Substantive contributions to the design, conduct, interpretation and reporting of the clinical trial will be recognised through the granting of authorship on the final trial report. The trial protocol, full study report, anonymised participant-level dataset and statistical code for generating the results will be made publicly available if all investigators agree to share the data.

## Data Availability

Not applicable; no datasets are included in this study protocol.

## Abbreviations

CRF: Case report form
CSF: Cerebrospinal fluid
CT: Computed tomography
EDH: Extradural haemorrhages
MRI: Magnetic resonance imaging
TBC-MUW: The Bioethics Committee of the Medical University of Warsaw
